# Scientific Advances

**Published:** 1889-05

**Authors:** C. C. Barker

**Affiliations:** Meriden, Conn.


### International Dental Journal.

Vol. X. May, 1889. No. 5.

### Original Communications.[1]

### SCIENTIFIC ADVANCES.

BY C. C. BARKER, MERIDEN, CONN.

That the microscope is a most efficient aid, and of undoubted utility in the modern attempts to solve the problems of life is certainly true.

You know that in the time of Mr. Lemuel Gulliver, it took a long sea voyage, and the perils of ship-wreck to reach Lilliput.

But now a trip to the world of little things is made, by means of this wonderful instrument, in safe and rapid transit.

In Gulliver's Travels an extended delineation is given of the Lilliputian fauna and flora, but his story is far exceeded by the botanical descriptions given by traveller after traveller concerning the marvellous plant life they have found within the realm of little things, now known as the bacterian world.

How concise is the report of their discoveries!

Let me give you a sample:

"Everywhere in the air of inhabited regions, in the water, and in the soil, are myriads of living organisms, which belong among the simple forms of life, and which are commonly called bacteria.

They are very minute, requiring high powers of the microscope to make them visible.

Some of them are capable of rapid multiplication, so that they may, under favorable circumstances, increase in number a thousand

[1] The editor and publishers are not responsible for the views of authors of papers published in this department, nor for any claim to novelty, or otherwise, that may be made by them. No papers will be received for this department that have appeared in any other journal published in this country. The journal is issued promptly on the 15th of the month.

or a million fold even, in a few hours. Some of these organisms appear, to be perfectly harmless having no effect whatsoever, so far as known, upon the health of animals and man.

Others are of positive value in preserving a healthful sanitary condition of the water, soil and air; which they do by breaking down and feeding upon organic compounds which might prove deleterious to health.

On the other hand, it has been definitely proven that a certain number of fatal diseases in man, and several in animals, are alone caused by the entrance into the body of special forms of bacteria.

These bacteria are often called "disease germs" and, like the bacteria which are harmless to man and animals, are very minute.

Thus the organism causing consumption, which is a little rod-like structure, is so minute that if placed end to end it would require from 4,000 to 6,000 of them to reach across the head of an ordinary sized pin."—T. Mitchell Prudden, M. D., (Eighth Annual Report Conn. State Board of Health, 1885).

Others tell us of the size and weight of these organisms.

Think of it! Is it not marvellous that things so exceedingly minute can be made appreciable to our vision?

But the ordinary lenses employed in histological work will not do it. "We must have the recent oil immersion lens,"—so says Dr. Black.

Yes,—and they classify these almost infinitesimal plants.

They tell of four genera.

1st. Sphero-bacteria or globular forms.

2d. Micro-bacteria, or minute rods.

3d. Desmo- or filo-bacteria.

4th. Spiro-bacteria, twisted, or spiral filaments.

This is the classification made by Cohn, another designation of the four genera is :

1st. *Micrococci* (the word means little grains) are round.

2d. *Bacteria*, proper. Very short cylinders.

3d. *Bacilli* are longer.

4th. *Spirilla* (Lucins Pitkin) twisted rods.

Each genus is also subdivided into varieties and species to many of which are given immense names which, in numerous instances, have a decided hydrocephalic flavor.

To ordinary minds it was a daring attempt thus to identify, classify and group them.

Was there a ever a subject so minute, and yet so broad, or larger?

But this is not all—reporter has followed reporter.

We have been told of their great variety, and most rapid and astonishing increase; how they swarm about us at every identical point of the compass, constantly threatening an invasion and attack upon the sacred citadel of life; indeed, it has come to be quite dogmatically asserted, that every disease to which human flesh is heir is produced by some special bacterium.

For something like ten years, there has been a perfect whirlwind of announcements and assertions in this regard, which has produced an intense mental ferment within the medical profession; until, with many, pathology and bacteriology have become almost synonymous terms. Placed amid such dangerous surroundings it is indeed surprising that humanity has survived until this late date.

We certainly ought to thank our lucky stars.

I cannot help sharing in some measure the skepticism of the Irish bishop, who after reading Gulliver's Voyage to Lilliput, and his travels there, remarked that, " There were some things in it which he could not believe."

But seriously one cannot withold a high respect for the experiments conducted with such careful precision in the laboratory established and presided over by Dr. Robert Koch, in Berlin. My information is obtained through the investigation and report of Dr. Prudden, made at the request of the Conn. State Board of Health; who declared it to be " probably the most complete and perfect which exists."

I cannot attempt a detailed description; suffice it to say that there they claim to identify and isolate single species—making pure cultures of them—studying their life history, and establishing whether " they are, or are not disease-producing—pathogenic."

They further claim that specific micro-organisms are present in " splenic fever, relapsing fever, erysipelas, tuberculosis, cholera, pneumonia, etc., etc., a long list. But these claims are by no means granted on every hand, many eniment medical men are taking strong exceptions to such radical views, and are sternly calling a halt to what they consider rash theorising.

The paper read at Washington before the International Medical Congress, in general session, by Prof. Semmola, of Naples, Italy, was strong and notable in this respect.

Let me give a few extracts :—

"These discoveries are proclaimed on all sides as the true foundation stones of biology, and doctors hasten with eagerness to proclaim that the only end for cure in sickness is inoculation of microbes, or at least, to prepare the organism for surviving the work of the cure by inoculation."

"Observers are too ready to assert that diseases following inoculations are produced by the germ; and I appeal to all lovers of truth to determine, if possible, which diseases are dependent upon inoculation and which are not."

"Who has seen malaria, or diphtheria, or any disease in which it was *proven* that the disease depended upon the microbe?"

"Rodents, ruminants and some other living organisms show predilection for anthrax; but the carnivora are exempt from many diseases caused by germs."

"Doraine and Koch failed in some attempts to transfer certain germs from one animal to another. What positive demonstration can they furnish of the causation of diseases?"

"Our physical and chemical knowledge of the blood of different animals is so small, that we have no means of knowing what animal furnishes a suituable soil for the growth of these microbes."

"The chemistry of the tissues of an animal is the true basis of judgment of its inoculability or non-inoculability by germs. This, I think, no scientific man will doubt."

"Man lives surrounded by micro-organisms, over which he triumphs, as he does over the material world."

"I have found by analysis the air full of microbes, but as yet we possess no test as to their being injurious or harmless germs. We cannot ascertain correctly the soil in which microbes grow, nor their mode of life, and hence one factor of the problem remains unknown."

"Diseases are produced by inoculation; but are these morbid processes dependent upon the germ or upon the soil in which it is implanted? Demonstration by this method fails; nothing is positively proven; and in the majority of cases common good sense pronounces against the attempted proof. If this method be correct, physiology must be suppressed or reversed. Suppose a man, when sweating, should enter a cave and remain there several hours, contracting a violent acute rheumatism, do you mean to say that I must seek the cause of the disease in a microbe? or did a profound derangement of his physiological conditions occasion the disease?"

Semmola's address was a long one, and bristling with strong points.

It is found in the *Medical News* of September 10, 1887, also in vol. 1, Trans. of the Ninth International Medical Congress.

In the "Anniversary address before the Medical Society of the State of New York," delivered at Albany February 8, 1888, by the President, Alfred L. Loomis, M.D.

Two of the four problems which he discussed and which he declared awaited solution were :—

1st. The relation of microbes to disease processes.

2d. Inoculation as a means of modifying or preventing disease.

His words were intelligent and instructive.

Among them were these:

"When we consider the millions of microbes by which we are constantly surrounded,that the soil beneath us, and the air around around us is laden with them, that they enter the body in the very air we breathe, the food we eat, the water we drink, and yet their influence is baneful only under certain conditions; that it is even possible for men to live in an atmosphere and surroundings which are ladened with the most pernicious form of bacterial life, and yet be perfectly well, the question comes to us, why is this? If the question is ever answered, it will not be until we have a fuller knowledge of the conditions of life, and the inherent properties of even the most carefully studied varieties of bacterial life."

From this language, it is evident that Dr. Loomis considers the theories of the bacterian pathologists as far from demonstrated.

Dr. Ezra M. Hunter, Secretary of the New Jersey State Board of Health, in an article written for the *Medical News* of March 17, 1888, and entitled, "Notes on Progressive Studies in Etiology, and Especially as to Micro-organisms as Sole factors of Disease," remarks that,

"Physicians generalize much more rapidly than do most painstaking specialists in so minute inquiries. Although ten or more years have passed, we may well remember that it was Cohn who says:

"So long as the makers of microscopes do not place at our disposal much higher powers, and as far as possible without immersion, we will find ourselves in the domain of the bacteria in the situation of a traveller who wandered in an unknown country at the hour of tranquility where the light of day no longer suffices to distinguish objects, and when he is conscious that, notwithstanding all his precautions, he is liable to lose his way."

Magnin closes his treatise (1880) thus:

"As to their *rôle* (micro-organisms) in fermentations, in putrefactions, in contagious diseases, and in surgical lesions, notwithstanding the considerable number of labors of which bacteria have been the objects in the different points of view, it is not yet possible to define in a certain manner."

In 1886, E. Klein opened his excellent manual on micro-organisms and disease by cautioning us against taking:

"As proved what has really not passed beyond the stage of possibility."

Prof. DeBarry, in the recent edition of his lectures (October, 1887), states similiar views with great precision, and with many cautions against too rapid scientific or clinical deductions.

Says Prof. DeChanmont (1887):

"Those who have followed the developements of the (microbe) question during late years, know what extraordinary studies have been made, and yet we are, as it were, merely hovering about the gate, and have barely entered the field at all."

Again:

"It is shown that pathogenic bacteria (or what are believed to be so), propagate best under unhygienic conditions, light, fresh air, and pure water being inimical."

Dr. Hunter adds here very positively:

"It behooves us to give greater consideration *to that class of causes which are generally most decisive of results.*"

Other prominent authorities are quoted—and last—but by no means least—are Dr. Hunter's own conclusions distinctly summarized.

Allow me to give two or three:

(f.) "It was only after much had been said of the micro-organisms of disease that we came to realize that the *rôle* played in the economy of nature, or of animal life by micro-organisms is *essential* for the maintenance of life. Hence, it was almost a surprise when we reached the term, *harmless bacteria*; and still more the term, *indispensable bacteria.*"

(g.) "Microbes have so much to do with what are, after all, chemical processes, that we are not to so far accept biology as to overthrow chemical metabolisms of disease. The biological facts still leave a study of disease from the standpoints of physiological and pathological chemistry. Germane to this is the recent paper of M. Vignal to the Academie des Sciences, published in *La France Medicale*, No. 99, an article in which not only are the number and variety of microbes in the alimentary canal noted, but the remarkable or different powers of chemical metabolism which they exercise. The claims of Pasteur for the wonder workings of microbes in the usual processes of digestion and their necessity are in the same line."

(h.) "A new field of inquiry opens as we study the *products* of micro-organisms, and the poisonous products of decomposition. As to either of these, we do not yet know all the relations that are biological or that are chemical. Thus, in anthrax, it is not the bacillus, but, as Hoffa and others have shown, "the poison products accumulating in the blood that causes the fatal issue."

So there is claimed to be a cholera ptomaine. See, also, the researches of Brieger on the tetanus bacilli, and of Rosenbach as to the two alkaloids, tetanin and tetanotoxin, both of which introduced into animals (without the bacillus) produce their specific action. So in the various ptomaines (selmi) and leucomaines we have to deal, perhaps with chemical products, and not with actual animal or vegetable life *in situ.*

In fact septic poison, or sepsin, results from various chemical processes which in themselves are destructive to every living micro-organism.

It is very plain to be seen that upon this exceedingly minute, and also exceedingly large subject, there is a great conflict of opinion and of doubt.

Let me say this—that if those chemical changes which we denominate as fermentation and putrefaction, are wrought by the instrumentality of these bacterian bits of plants—and *alone* by them—if this be established as impregnable fact, then all past theories of disease must sooner or later be greatly modified.

The prognosis of this agitation is favorable.

The present condition is symptomatic of those mental throes which precede the birth and reception of new truth.

Great and wonderful benefits will as surely come as day dawns after the night.

Their benefits have already begun to appear to dental practitioners.

We, as dentists, have reason for congratulations that we have among us men who have commenced their investigations on this subject at foundation lines, and from those initial steps have demonstrated certain facts which are already becoming the basis of a more intelligent practice.

With their investigations and experiments you are all more or less familiar.

Whatever may come to be considered as the true relation of micro-organisms to general disease, by the medical practitioner—there is no room for doubt that they are proven factors in the production of dental caries.

Let me ask in conclusion:—

Does it not, in the presence of these wonders, require vastly more credulity to reject the conviction that we dwell within the realm of the Infinite One—the Almighty Father of life, light and truth—than it does reverently to yield our faith?

I think so.

Herbert Spencer's name is commonly placed upon the list of Agnostics, and yet, I find he has said:

"Amid the mysteries, which become the more mysterious, the more they are thought about, there will remain the one absolute certainty, that we are ever in the presence of an infinite and eternal energy, from which all things proceed."

